# Kinase Domain Insertions Define Distinct Roles of CLK Kinases in SR Protein Phosphorylation

**DOI:** 10.1016/j.str.2008.12.023

**Published:** 2009-03-11

**Authors:** Alex N. Bullock, Sanjan Das, Judit É. Debreczeni, Peter Rellos, Oleg Fedorov, Frank H. Niesen, Kunde Guo, Evangelos Papagrigoriou, Ann L. Amos, Suhyung Cho, Benjamin E. Turk, Gourisankar Ghosh, Stefan Knapp

**Affiliations:** 1 Structural Genomics Consortium, University of Oxford, Old Road Research Building, Roosevelt Drive, Oxford OX3 7DQ, UK; 2 Department of Chemistry & Biochemistry, University of California, San Diego, La Jolla, CA 92093, USA; 3 Department of Pharmacology, Yale University School of Medicine, New Haven, CT 06520, USA; 4 Department of Clinical Pharmacology, University of Oxford, Old Road Research Building, Roosevelt Drive, Oxford OX3 7DQ, UK

**Keywords:** SIGNALING

## Abstract

Splicing requires reversible phosphorylation of serine/arginine-rich (SR) proteins, which direct splice site selection in eukaryotic mRNA. These phosphorylation events are dependent on SR protein (SRPK) and cdc2-like kinase (CLK) families. SRPK1 phosphorylation of splicing factors is restricted by a specific docking interaction whereas CLK activity is less constrained. To understand functional differences between splicing factor targeting kinases, we determined crystal structures of CLK1 and CLK3. Intriguingly, in CLKs the SRPK1 docking site is blocked by insertion of a previously unseen helix αH. In addition, substrate docking grooves present in related mitogen activating protein kinases (MAPKs) are inaccessible due to a CLK specific β7/8-hairpin insert. Thus, the unconstrained substrate interaction together with the determined active-site mediated substrate specificity allows CLKs to complete the functionally important hyperphosphorylation of splicing factors like ASF/SF2. In addition, despite high sequence conservation, we identified inhibitors with surprising isoform specificity for CLK1 over CLK3.

## Introduction

Cdc2-like kinases (CLK) are conserved throughout eukaryote evolution, from *Saccharomyces cerevisiae* (KNS1), *Arabidopsis thaliana* (AFC1-3), and *Drosophila melanogaster* (DOA) to mouse (CLK/STY) and human (CLK1-4) (Yun et al., 1994). Based on an unique and conserved “EHLAMMERILG” signature motif, CLKs are also often referred to as “LAMMER kinases.” The conserved signature motif of CLKs has been suggested to dictate CLK substrate specificity (Yun et al., 1994). Interestingly, CLKs are dual-specificity kinases, which have the ability to autophosphorylate at tyrosine residues but phosphorylate their substrates exclusively on serine/threonine residues (Nayler et al., 1997).

Consequences of CLK dysfunction are dramatically illustrated in *Drosophila* by defects in the CLK homolog DOA (darkener of apricot). Mutations in the *doa* locus affect sexual differentiation by specifically disrupting sex-specific splicing of *doublesex* (*dsx*) pre-mRNA through interaction with the transformer splicing regulatory proteins TRA and TRA2 (Du et al., 1998). The *doa* gene has an essential role in *Drosophila* embryogenesis; it is expressed throughout development and its mutation leads to defects in segmentation, eye formation, and neuronal development (Yun et al., 1994). In humans, splicing of the homolog hTRA2beta is regulated by CLK2 (Stoilov et al., 2004) whereas CLK1 plays an important role in neuronal differentiation (Myers et al., 1994). CLK3 is abundantly expressed in mature spermatozoa and might play a role in the fertilization process (Menegay et al., 1999).

Alternative splicing is controlled by phosphorylation of serine/arginine-rich (SR) splicing factors, which have been identified as CLK interaction partners and substrates (Colwill et al., 1996a, 1996b; Duncan et al., 1997). Splicing is an important regulatory mechanism in eukaryotes that allows a single gene to generate multiple protein isoforms with distinct function. Indeed, some 35%–60% of human genes encode at least two alternatively spliced isoforms (Modrek and Lee, 2002), and deregulation of splicing is frequently linked to hereditary diseases (Faustino and Cooper, 2003).

The prototypical SR protein ASF/SF2 contains two RS (arginine/serine) repeat domains with multiple arginine-serine dipeptides. Phosphorylation of the first RS domain (RS1) triggers nuclear import and accumulation in nuclear speckles (Ma et al., 2008; Velazquez-Dones et al., 2005). This phosphorylation event is controlled by SR-protein kinase SRPK. Subsequent phosphorylation of the RS2 domain by CLK dissolves these nuclear speckles and releases ASF/SF2 for splicing and dephosphorylation (Colwill et al., 1996b; Duncan et al., 1998). Selectivity for the RS1 domain of SRPK kinases can be rationalized by the crystal structure of the SRPK1-ASF/SF2 complex (Ngo et al., 2008). The first RS repeat is bound to an acidic docking groove within the kinase C-lobe formed by helix αG and a helical insert typically found in MAPKs. A self-primed phosphoserine from the C′ end of RS1 is also observed close to the active site at a probable P+2 site. Biochemical experiments have suggested that the entire RS1 domain is threaded in the C′ to N′ direction with the successive phosphorylation of each RS dipeptide driving their transfer to the basic P+2 pocket. Docking to the RS2 domain, however, mighty be disfavored by the presence of shorter interrupted stretches of RS dipeptides. CLK activity is not restricted to RS repeats, and these kinases might have more diverse substrates in splicing control (Colwill et al., 1996a).

In analogy with SRPK1, it has been suggested that the LAMMER motif forms a conserved docking site for CLK substrates. To assess this hypothesis and rationalize the distinct activities of the SRPK and CLK kinases regulating splicing, we determined the crystal structures of the human CLK1 and CLK3 kinase domains. Our structural analysis revealed that despite a similar MAPK insert and an αG helix comprising the LAMMER motif, the restrictive docking sites of both the SRPK and MAPKs are disrupted in the CLK family by two previously unseen insertions. We determined the substrate specificity of CLK1 and compared the two CLK isoforms with respect to their binding profiles versus a large panel of established kinase inhibitors. The identified chemical scaffolds and the presented high-resolution structure of CLK1 in complex with the ATP mimetic inhibitor hymenialdisine provide the basis for further structure-based inhibitor development and validation of the functional roles of CLK kinases in vivo.

## Results

### Overview of the CLK1 and CLK3 Structures

**Figure 1:**
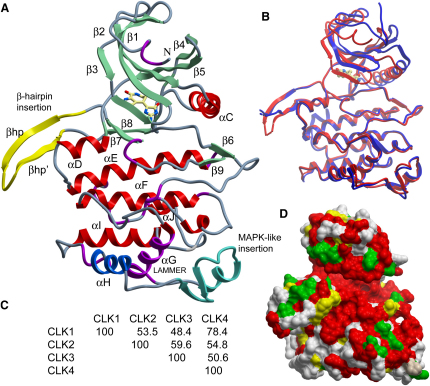
Overview of the CLK1 and CLK3 Structures (A) Structure of the CLK1 kinase domain. Specific structural insertions defining the LAMMER kinases are highlighted by different colors and are labeled. The inhibitor debromohymenialdisine is shown in stick representation. (B) Structural superimposition of the nonphosphorylated CLK1 (red) and CLK3 structures (blue). (C) Sequence identity among the members of the human CLK family. (D) Surface residue conservation mapped onto the CLK1 structure. Residues conserved among all CLK family members are shown in red, conserved hydrophobic residues in yellow, conserved polar residues in green and regions of diversity are shown in gray. A sequence alignment is shown in Supplemental Data.

The kinase domains of CLK1 and CLK3 were expressed in *E. coli* in active form as demonstrated by extensive autophosphorylation. Both recombinant proteins required optimization of buffer conditions for crystallization studies (see Experimental Procedures) and phosphatase treatment to obtain homogeneous sample. High-resolution crystal structures were solved for CLK1 in complex with the kinase inhibitor hymenialdisine and for the apo-CLK3 kinase domain (see Table 1 for crystallization and refinement statistics). Both structures were well defined by the electron density except for two short loop regions in CLK1 (residues 307–310 and 483–484) and three regions in CLK3 (residues 127–135, 304, and 482–484) that were not modeled. The catalytic domains of CLK1 and CLK3 display the typical protein kinase fold as shown in Figure 1. The N-terminal lobe consists of three β strands (β1–β3) followed by an α helix (αC) and two further β strands (β4 and β5). The C-terminal lobe exhibits several unique features that define the CLK family. The highly conserved signature motif of this group (EHLAMMERILG, residues 386–396 in CLK1 and 381–391 in CLK3), from which the family name LAMMER-kinases is derived, forms part of helix αG located at the bottom of the C-terminal lobe (Figure 1A) and is made solvent-inaccessible by a large insertion between residues 400 and 432 in CLK1 and 395 and 427 in CLK3. This region displays a high similarity to the typical helix-loop-helix insertion motif found at the same site in the MAPK family. However, in the CLK structures, the second helix is replaced by a small two-strand β sheet (formed by residues 409–418 in CLK1 and 404–413 in CLK3). This motif is followed by helix αH that is unique to these CLK structures (residues 424–432 and 419–427 in CLK1 and CLK3, respectively). A second unique insertion conserved in the CLK family is present at the top of the C-terminal lobe where residues 300–317 in CLK1 and 295–312 in CLK3 form an extended β-hairpin (βhp-βhp′).

### Structural Differences between CLK1 and CLK3

Superposition of all main chain atoms of CLK1 and CLK3 reveals high structural similarity between the two proteins (Figure 1B). The main structural difference is a change in the relative orientation of the N- and C-terminal lobes such that CLK3 exhibits a more open conformation than CLK1. This change is reflected in the root-mean-square deviation (rmsd) values, which are 1.72 Å for all main chain atoms but just 0.98 Å and 0.97 Å for the individual N- and C-terminal lobes, respectively. The difference in lobe orientation is likely to be an effect of the inhibitor bound to CLK1 mimicking binding of the ATP cofactor. As highlighted in Figure 1B, the most divergent regions are the MAPK-like insertion, helix αG, and the partially disordered tips of the βhp-βhp′ hairpin insertion. CLK3 also shows additional secondary structure elements at the N and C termini not observed in CLK1: first, a short hairpin comprising residues 136–145 that folds above the N-terminal domain; and second, an additional helical segment at the C terminus formed by residues 476–481. Within the CLK family, CLK1 and CLK3 are the most distantly related with respect to a sequence alignment (Figure 1C; see Figure S1 available online). Both proteins share only 48.4% sequence identity. However, many of the surface residues that are conserved throughout the CLK family are the same in CLK1 and CLK3 (Figure 1D).

### Active Conformation of the Catalytic Domain

**Figure 2:**
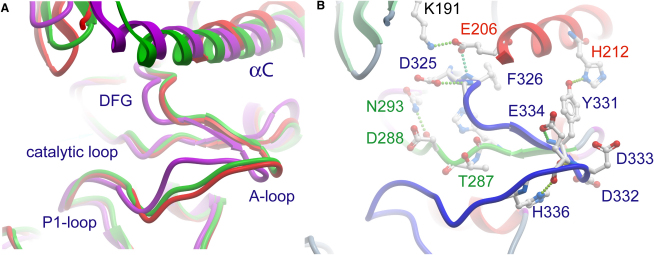
CLK Activation Segments (A) Superimposition of the activation segments of CLK1 (red) and CLK3 (green) as well as of SRPK1 (magenta) (PDB code 1WBP). The functional elements of the segment (DFG, A loop and P1 loop) as well as the catalytic loop and the helix αC are labeled. (B) Activation segment of CLK1. The activation segment is shown in blue, the catalytic loop in green, and αC in red. Selected residues within these structural elements are labeled in corresponding colors.

The unphosphorylated CLK catalytic domains are in an active conformation as indicated by a closed conformation of both kinase lobes and well-ordered activation segments (Figure 2). In both domains, helix αC is positioned in close proximity to the ATP binding site with the characteristic salt bridge between the conserved αC glutamate (CLK1, Glu206) and the active site lysine (Lys191). The activation loop (A loop) of both kinases is well defined in the electron density and adopts the characteristic conformation of active kinases despite the absence of a phosphorylation. Its conformation is similar in both CLK structures, mainly constrained by a short antiparallel β sheet formed between β6 and β9 and a conserved hydrogen bond between His336 and Glu334 in CLK1. A similar A loop conformation is also found in SRPK1 (Ngo et al., 2005). Both CLK activation segments have a very acidic A loop stabilized by polar contacts. An additional hydrogen bond is formed in CLK1 between the A loop residue Tyr331 and His212 in αC. This interaction, however, is not conserved: in CLK3 it is replaced by a hydrophobic contact between the Tyr331-equivalent Phe326 and the αC residues Val204 and Lys207. The catalytic loop motif HRD is replaced by a HTD motif among all members of the CLK family and in SRPK1.

### Unique LAMMER Kinase Insertions

**Figure 3:**
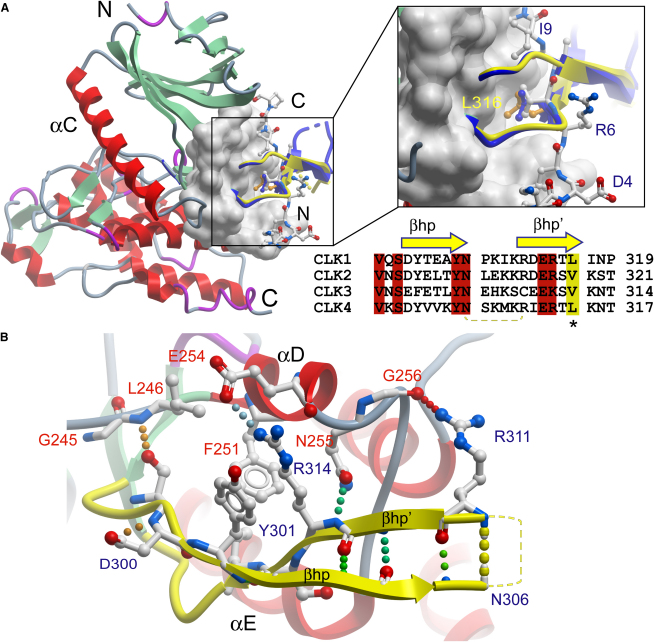
CLK β-Hairpin Insert (A) Overview showing the location of the β-hairpin insert conserved within the CLK family. The hairpin interacts with a groove formed by helix αD and αE. Shown is the CLK1 β-hairpin (βhp and βhp′) in yellow and the CLK3 β-hairpin in blue. A docking peptide of the MAPK p38 (PDB code 1LEW) is shown in ball-and-stick representation (Chang et al., 2002). A detailed view of the hydrophobic interaction formed by Leu316 conserved within the CLK family (CLK3 is shown in blue). Leu316 superimposes well with a leucine present at this position in the MEF2A-derived MAPK docking peptide (colored in white). A Sequence alignment of human CLK family β-hairpin inserts is shown under the peptide interaction insert. Conserved residues are highlighted in red, the conserved hydrophobic residues making contact with the binding groove are highlighted in yellow and marked by an asterisk. The disordered tip of the hairpin insert is shown by a dotted yellow line. (B) Interactions formed by CLK1 β-hairpin residues with the surface of CLK1. Residues present in the hairpin are labeled in dark blue and those present in the kinase lower lobe are labeled in red.

The most striking feature of the CLK C-terminal lobe is a long insertion between the two sheets β7 and β8. This insert forms the CLK-specific βhp-βhp′ hairpin that folds over a shallow groove created by the helices αD and αE (Figure 3). Interestingly, substrates of MAPKs recognize the same shallow groove through so-called D motif docking sites by using mainly hydrophobic contacts. Although MAPK docking peptides bind in an orientation that is rotated approximately 90° compared with the β-hairpin, their central hydrophobic patch is mimicked by a conserved hydrophobic residue in the β-hairpin (Figures 3A and 3B). This β-hairpin structure is common to CLK1 and CLK3. Although there is low sequence homology in the region, a number of positions in the β-hairpin are conserved throughout the CLK family (Figure 3C). Apart from the hydrophobic patch (Leu316 in CLK1) and a conserved valine (Val297), the β-hairpin is docked to the shallow groove by a number of hydrogen bonds, including backbone contacts formed by the side chains of Arg311 and Ser299 in CLK1. This hairpin is also the site of a structurally uncharacterized insertion of ∼250 residues in SRPK1.

**Figure 4:**
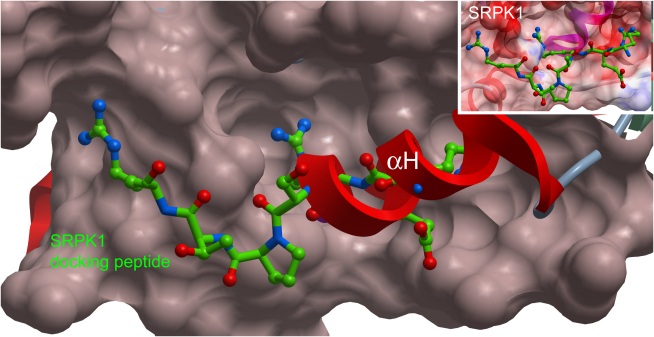
Docking Grooves and Determinants of Substrate Binding of CLK1 and SRPK1 Shown is the surface of SRPK, the corresponding docking peptide and secondary structure elements for CLK1. The SRPK docking site is blocked by helix αH in CLK family members. The docking site in SRPK is important for substrate recognition (Ngo et al., 2008). In this figure, CLK1 has been superimposed onto the structure of SRPK1 in complex with a docking peptide derived from the yeast SR-like protein Npl3p (PDB code 1WBP; Ngo et al., 2005). The superimposition showed that binding of a potential docking peptide is impaired due to the presence of helix αH in CLK family members. Inset shows SRPK1-docking peptide alone.

The crystal structure of SRPK1 revealed an unusual docking groove in the C-terminal lobe (formed between helix αF and helix αG) that is not present in the CLK structures, thus providing a potential explanation for the restriction of SRPK1 activity toward RS substrate regions. This site is located adjacent to the MAPK-like insertion, which is present in both SRPK1 and the CLKs. However, the structural equivalent of the docking groove in SRPK1 is covered by the unique CLK helix αH (Figure 4). Surprisingly, this helix also buries helix αG, which comprises the highly conserved EHLAMMERILG signature motif (Figure 4). On the basis of sequence alignments, it has been suggested that this motif is solvent exposed and on the basis of its extraordinary conservation the signature motif has been predicted as an interaction site for either substrates or effectors (Yun et al., 1994). However, in both CLK structures the LAMMER motif is made solvent-inaccessible by αH and the adjacent MAPK-like insertion. Most notably, the alanine (uniquely replaced by valine in CLK3) that lies at the center of helix αG is packed against a strictly conserved tryptophan (CLK1, Trp419; CLK3, Trp414) located between the αH and the MAPK-like inserts.

### Determination of Substrate Specificity

**Figure 5:**
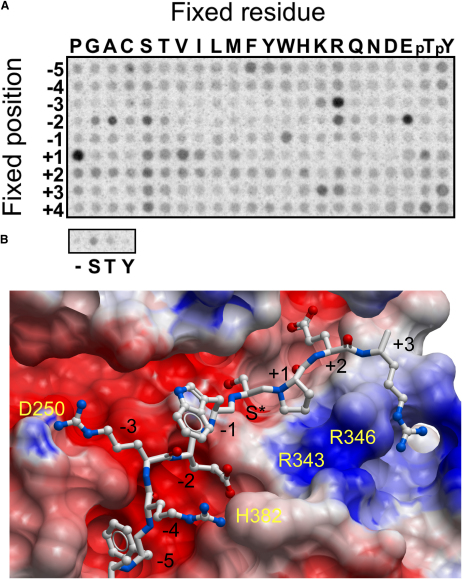
Consensus Phosphorylation Motifs (A) Biotinylated peptides bearing the indicated residue at the indicated position relative to a central Ser/Thr phosphoacceptor site were subjected to phosphorylation by CLK1 using radiolabeled ATP. Aliquots of each reaction were subsequently spotted onto a streptavidin membrane, which was washed, dried, and exposed to a phosphor screen. (B) Model of a CLK1 consensus peptide complex. The consensus sequence was modeled into the CLK1 substrate binding site using the structure of the PKA substrate complex as a template (PDB code 1JBP). CLK1 surface residues cited in the text are labeled in yellow and substrate positions are labeled in black.

Consistent with its active conformation, the catalytic domain of CLK1 showed constitutive kinase activity against a degenerate peptide array library. We determined the active site mediated substrate specificity of CLK1 and compared its specificity with previous data reported for CLK2 and CLK3 (Figure 5) (Hutti et al., 2004; Miller et al., 2008). CLK1 showed an approximately two-fold phosphorylation preference for serine over threonine and no detectable phosphorylation of tyrosine residues. The primary selectivity for CLK1 was for arginine in the position P-3 (3 residues N-terminal to the phosphorylation site), which was also observed for CLK2 and CLK3. CLK1 also showed a strong selection for proline at position P+1, whereas CLK2 and CLK3 were less selective at this position. Interestingly, CLK1 lacks selectivity for arginine at position P+1 as seen for CLK2 and CLK3 and that has also been noted in previous studies of LAMMER kinases (including *Drosophila* DOA) (Nikolakaki et al., 2002). A greater role for CLK3 as a SR-specific protein kinase is also suggested by its stronger preference for arginine in P-1, whereas CLK1 shows no preference at this position. Although all CLK kinases disfavor large hydrophobic residues at P-2, CLK1 showed a pronounced preference for glutamate at that position.

To rationalize the observed substrate specificity for CLK1, we generated a docking model of a substrate complex using the peptide cocrystal structure of PKA (Protein Data Bank [PDB] code 1JBP) as a template (Figure 5B). The model explains the main selectivities observed in the peptide array experiment. In particular, the proline residue in P+1 fits in a small hydrophobic surface pocket formed by hydrophobic regions of the Arg346 side chain. The strong preference for glutamate in P-2 can be explained by a hydrogen bond interaction with His382, and the selection for arginine in P-3 is likely to be due to formation of a salt bridge with Asp250. Also, the preference for phenylalanine in position −5 can be explained by a deep hydrophobic surface cavity. However, it is unlikely that the conserved CLK1 residues Arg343/Arg346 select for a phosphorylated peptide in P+2 as suggested for SRPK1 (Ngo et al., 2008). This basic pocket is too distant from this substrate position and the side chain is oriented away from this basic binding surface pocket.

### ASF/SF2 Phosphorylation by CLK1

**Figure 6:**
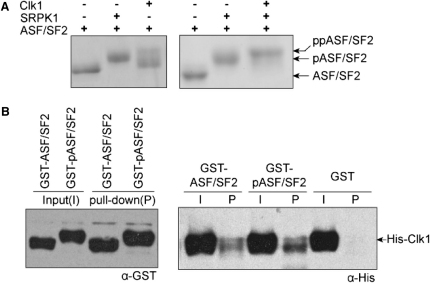
CLK1 and SRPK1 Show Nonoverlapping Complementary Activity (A) Coomassie-stained SDS-PAGE gel showing that CLK1 efficiently converts pASF/SF2 into ppASF/SF2. (B) GST pull-down assay showing association of CLK1 with ASF/SF2 and pASF/SF2.

To further characterize the role of CLK1, we analyzed the activity of the full-length protein against ASF/SF2. The additional N-terminal domain in CLK1 is required for efficient ASF/SF2 binding. The N-terminal domain resembles an RS domain with multiple arginine-serine dipeptides. As a kinase-inactive mutant, the full-length protein colocalizes with other splicing factors to nuclear speckles, whereas wild-type CLK1 causes speckles to dissolve (Colwill et al., 1996b; Duncan et al., 1998). According to the current model, SRPK1 hypophosphorylates ASF/SF2 (generating pASF/SF2), which causes its targeting to nuclear speckles. Subsequently pASF/SF2 becomes hyperphosphorylated by CLK1 and is released as ppASF/SF2. In our experiments, SRPK1-treated ASF/SF2, which contains 12–14 phosphorylated serines, was used as a control to monitor the extent of ASF/SF2 phosphorylation by CLK1. ASF/SF2 treated with CLK1 alone produced two dominant phosphorylated species; the major product migrated faster than pASF/SF2, indicating fewer than 12 phosphates, whereas the minor product, representing ppASF/SF2 (hyperphosphorylated) migrated more slowly (Figure 6A). However, treatment with both CLK1 and SRPK1 completes phosphorylation (Figure 6A). These results are consistent with the nonoverlapping substrate specificity of the two kinases and the loss of selection of CLK1 for the RS1 repeat. Indeed, using glutathione S-transferase (GST) pull-down experiments we observed binding of CLK1 to both unphosphorylated protein and pASF/SF2, with the robust binding to pASF/SF2, supporting the complementarity of the two kinases (Figure 6B).

### Inhibitor Binding and Specificity

**Figure 7:**
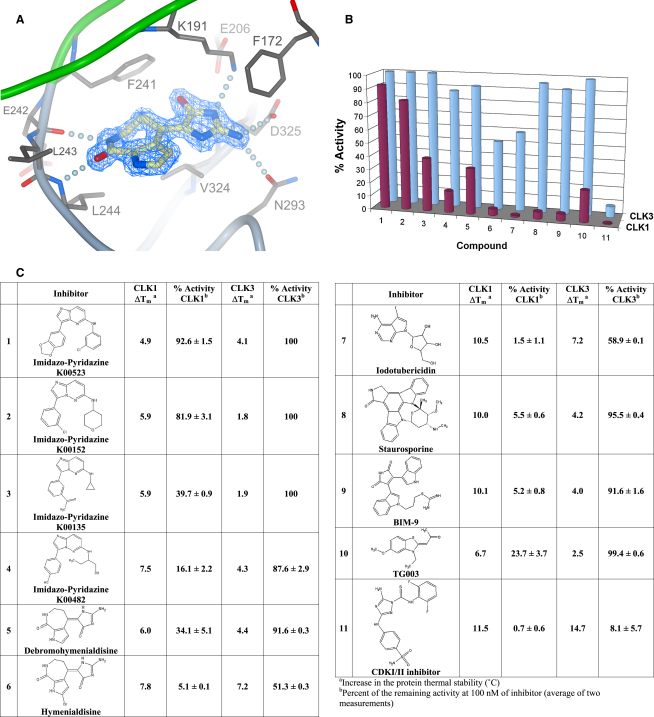
Inhibitor Binding and Specificity (A) Binding of debromohymenialdisine to CLK1. Electron density (2FoFc) for the inhibitor is shown in blue. (B) Chart showing CLK1 and CLK3 kinase activities relative to the reference in the presence of 100 nM of 11 compounds causing the highest T_m_ shifts. (C) Table of the inhibitor data with corresponding chemical structures.

CLK1 was crystallized in the presence of hymenialdisine, a marine sponge metabolite that has nanomolar activity against CDKs, MEK1, GSK3β, and CK1 (Meijer et al., 2000). In the crystal structure, hymenialdisine is bound in the typical ATP-mimetic manner, at the hinge backbone region where residues Glu242, Leu243, and Leu244 interact through one water-mediated and two direct main-chain hydrogen bonds. In addition, further hydrogen bonds are observed with conserved residues Asp305, Lys191, and Asn294 resembling the well-conserved binding mode of this inhibitor class (Figure 7A). No density was observed for the bromide substituent, suggesting that the halogen bond was hydrolyzed to yield debromohymenialdisine.

Previous work identified the benzothiazole compound TG003 as a CLK1-specific inhibitor in vitro with an IC_50_ of 20 nM (Muraki et al., 2004). Such inhibitors provide interesting biological tools as well as potential drugs. We used a facile thermal stability shift assay to rapidly screen a collection of several hundred commercially available kinase inhibitors including TG003. This method identifies binding compounds by a shift in the protein's melting temperature, T_m_. For a large number of kinase inhibitors, these values have proven to correlate highly with binding constants determined by direct measurement (e.g., isothermal titration calorimetry) or with IC_50_ values such that larger T_m_ shifts are observed for higher affinity inhibitors (Bullock et al., 2005). A recent systematic study of 60 protein kinases and over 150 validated kinase inhibitors indicated that a 4°C shift in thermal stability at an inhibitor concentration of 10 μM corresponds to a binding affinity below 1 μM whereas an 8°C shift typically reflects a *K*_D_ of 100 nM or less (Fedorov et al., 2007). From our screening of CLK kinases, we selected 11 compounds with a T_m_ shift greater than 4°C for further analysis of their kinase inhibitory activity at 100 nM concentration (Figure 7). Overall, CLK1 appears more susceptible to inhibition than CLK3. CLK1 showed less than 50% activity with 9 of the 11 inhibitors compared with just one with CLK3. This dual-specificity inhibitor, compound 11 (a well-characterized CDK1/2 inhibitor), was the most effective compound for both kinases, reducing CLK1 activity to below 1%. Six CLK1 inhibitors were identified with greater potency than TG003. However, five of these include the more promiscuous staurosporine, BIM-9, hymenialdisine, iodotubericidin, and the CDK1/2 inhibitor. The final inhibitor in this group, compound 4, belongs to a novel class of imidazo[1,2-b]pyridazines, which are more selective and currently being investigated as a promising inhibitor scaffold for the development of antioncogenic PIM kinase inhibitors (Pogacic et al., 2007).

## Discussion

CLK and SRPK kinases mediate processive hyperphosphorylation of SR proteins that direct RNA splicing, but show distinct binding activities and kinetics (Ma et al., 2008; Velazquez-Dones et al., 2005). Understanding of SRPK activity has been advanced by recent crystal structures of the SRPK1 kinase domain, which revealed an unusual docking site restricting SRPK1 activity toward the first RS repeat domain (RS1) of ASF/SF2. Here the determined structures and substrate specificity of CLK1 provide corroborative evidence to support the less constrained activity of CLK kinases.

Both the SRPK and CLK kinases show a constitutively active conformation and share a similar variation of the MAPK insert that is a feature of the CMGC family. Most revealing, the docking groove of SRPK is lost in the CLK1 and CLK3 structures due to the previously unseen insertion of helix αH. This buries the signature EHLAMMERILG motif of helix αG that defines the subfamily of LAMMER kinases. Rather than forming the expected substrate interaction site, this sequence conservation appears to reflect its important role within the structural core of the kinase C-lobe. In addition, the typical MAPK docking site is also occupied by the unusual βhp-βhp′ hairpin insertion, which forms a similar hydrophobic patch with the kinase. The much larger domain insertion present at the same site in SRPK1 is not required for substrate interaction or catalysis, but is suggested to stabilize the active conformation (Ngo et al., 2007).

SRPK kinases are known to be highly specific for RS repeat substrates and to only phosphorylate serine (not threonine) residues that are located adjacent to arginines (and not lysines) (Gui et al., 1994; Wang et al., 1998). In contrast, CLK kinases have unusual dual specificity with propensity to autophosphorylate on serine, threonine, and tyrosine residues (Ben-David et al., 1991; Howell et al., 1991). In our degenerate peptide substrate arrays, the purified CLK activity was preferentially targeted toward serine rather than threonine residues, but tyrosine substrate phosphorylation was not observed. CLK1 behaved as a typical CDK-like proline-directed kinase with strong selectivity for serine phosphorylation sites with proline at position P+1 and arginine at position P-3, whereas CLK2 and CLK3 showed intermediate specificity compared with SRPK1 (Miller et al., 2008). These results correlate well with the substrate phosphorylation sites of CLK1 observed in vivo (Colwill et al., 1996b). For example, CLK1 activates PTP1B by serine phosphorylation within this motif (Moeslein et al., 1999). In ASF/SF2 the RS1 domain targeted by SRPK1 contains a segment of eight consecutive RS dipeptides, whereas the RS2 motif contains three SP dipeptides that interrupt the RS repeat. Our data support that CLK1 complements SRPK1 activity due to the preferential phosphorylation of this second motif. This is directed through (i) absence of the restrictive docking groove; (ii) selection for proline at position P+1; and (iii) colocalization to nuclear speckles directed by the N-terminal RS domain of CLK1.

Dysregulation of alternative splicing is a common feature of human cancers (Venables, 2004). ASF/SF2 is a newly identified proto-oncogene, found to be upregulated in a variety of human cancers; a slight overexpression of this protein is sufficient to transform immortal rodent fibroblasts giving rise to sarcomas in nude mice (Karni et al., 2007). The benzothiazole compound TG003 was recently reported to inhibit ASF/SF2-dependent splicing of β-globin pre-mRNA in vitro by suppression of CLK1-mediated phosphorylation (Muraki et al., 2004). It also suppressed CLK1-dependent alternative splicing in mammalian cells and rescued the embryonic defects induced by excessive CLK activity in *Xenopus*. Many other diseases are linked to specific splicing changes. For example, aberrant CLK2 splicing is associated with exon 10 inclusion in tau, which produces the neurodegenerative fibrillar aggregates found in Alzheimer's disease (Glatz et al., 2006; Hartmann et al., 2001). The high-resolution structure of CLK1 in complex with the kinase inhibitor hymenialdisine provides a basis for further structure-based inhibitor development. Such compounds will form valuable tools to probe CLK function and therapeutic potential. Of particular interest from our ligand screening is compound 4, a novel imidazo[1,2-b]pyridazine that shows potency and selectivity comparable with TG003.

Overall, the structures and substrate specificity profiles provide a rationale for the distinct activities observed for the CLK and SRPK kinase families. The CLK kinases show features of both the MAPK and CDK family kinases such as proline-directed activity (particularly for CLK1). Their structures reveal that the LAMMER kinase family-specific insertions block known interaction sites and, thus, promote target selection by the active site to complement SRPK kinases. These data further our understanding of the interplay of these two kinase families in alternate splicing and reveal possible intervention points for cancer treatment.

## Experimental Procedures

### Protein Expression and Purification

The kinase domains of human CLK1 (residues: 148–484 [C terminus, gi: 4758008]) and CLK3 (residues 127–484, gi: 153791372 isoform B) were subcloned by ligation independent cloning into a PET-derived expression vector, pLIC, and expression performed in BL21(DE3) with 1 mM IPTG induction for 4 hr at 18°C. These constructs were identified after expression screening nine different N- and C-terminal truncation constructs for each kinase. Cells were lysed using a high-pressure homogenizer, cleared by centrifugation, and the lysates purified by Ni-NTA chromatography. The eluted proteins were treated with lambda phosphatase together with TEV protease overnight to remove phosphorylation and the hexahistidine tag, respectively. The proteins were further purified by size exclusion chromatography using a S75 16/60 HiLoad column. A fluorescence-based thermal stability shift assay (Vedadi et al., 2006) was used to screen for optimal buffer conditions to prevent precipitation above concentrations of 1 mg/ml. The midpoint of the unfolding transition, T_m_, was shifted by the addition of L-arginine/L-glutamic acid by more than 10°C. The preferred buffer, 50 mM HEPES (pH 7.5), 500 mM NaCl, 5% glycerol, 50 mM L-glutamic acid, 50 mM L-arginine, increased solubility and resulted in monodisperse protein as judged by analytical ultracentrifugation. A final purification step using anion exchange chromatography with a MonoQ 5/50 GL column was used to resolve nonphosphorylated CLK3. The correct protein masses were confirmed by liquid chromatography electrospray ionization mass spectrometry.

### Determination of Peptide Phosphorylation Specificity

Phosphorylation motifs were determined by phosphorylation of a set of 201 peptide mixtures in multiwell plates essentially as described by Hutti et al. (2004). Reactions contained 20 mM HEPES (pH 7.4), 10 mM MgCl_2_, 1 mM dithiothreitol (DTT), 0.1% Tween 20, 50 μM ATP (including 0.3 μCi/μl γ-[^33^P]-ATP), 50 μM peptide substrate, and 1 μM CLK1 kinase for 2 hr at 30°C. Peptide substrates had the general sequence YAXXXXX-S/T-XXXXAGKK(biotin), where S/T is an equal mix of serine and threonine, and X represents degenerate positions comprising 17 amino acids (excluding cysteine, serine and threonine). Each of the 201 peptides in the set had 1 of the X positions fixed as 1 of 22 residues (all unmodified amino acids, phosphothreonine, or phosphotyrosine). To quantify the extent of phosphorylation of each peptide, aliquots were spotted onto a streptavidin membrane, which was washed, dried, and exposed to a phosphor screen as done previously (Hutti et al., 2004).

### Protein Stability Shift Assay

Thermal melting experiments were carried out using the Mx3005p real-time polymerase chain reaction machine (Agilent) and employing a protein concentration of 2 μM. The assay and data evaluation were carried out as described elsewhere (Fedorov et al., 2007; Matulis et al., 2005; Niesen et al., 2007). Imidazo-pyridazines were purchased from Biofocus (DPI), and all other inhibitors were purchased from Calbiochem.

### Kinase Assay

Phosphorylation reactions were monitored using a coupled-enzyme assay in which ADP production is coupled to NADH oxidation by pyruvate kinase (PK) and lactate dehydrogenase. The assay was carried out in a total volume of 200 μl in a buffer containing 50 mM HEPES (pH 7.5), 150 mM NaCl, 10 mM KCl, 2 mM DTT, 10 mM MgCl_2_, 1.0 mM phosphoenolpyruvate, 0.2 mM NADH, 15 U/ml pyruvate kinase, 30 U/ml lactate dehydrogenase, and 30 nM CLK1. The reaction was monitored at 340 nm at 25°C on a Spectramax spectrophotometer (Molecular Devices, Sunnyvale, CA). The reaction was started by addition of 0.1 mM ATP after a 10 min preincubation of the reaction mixture at 25°C. The CLK1 consensus peptide (AFRREWSPGKEAKK) was used as substrate at a concentration of 100 μM. Inhibitors, dissolved in dimethyl sulfoxide (DMSO), were added at the beginning of the pre-incubation period resulting in a final DMSO concentration of 2% in the assay. Kinetic analysis was performed by nonlinear regression fitting using the program KaleidaGraph (Synergy Software, CA).

### GST Pull-Down Assay

The phosphorylation of His-ASF/SF2 by SRPK1, CLK1, or both was performed in 20 mM Tris-HCl (pH 7.5), 75 mM NaCl, 1 mM DTT, 2 mM MgCl_2_, 0.1% NP40, 10% glycerol, 1 mM phenylmethanesulphonylfluoride (PMSF), 0.01 mg bovine serum albumin (BSA), and 1 mM ATP. The products were resolved in 10% SDS-PAGE.

Hypophosphorylation of GST-ASF/SF2 was carried out by incubating 1.5 ug GST-ASF/SF2 and 5 ug His-SRPK1(ΔNS3) in 20 mM Tris-HCl (pH 7.5), 75 mM NaCl, 1 mM DTT, 2 mM MgCl_2_, 0.1% NP40, 10% glycerol, 1 mM PMSF, and 0.01 mg BSA and 1 mM ATP for 2 hr at room temperature. Because SRPK1 tends to interact with pASF/SF2, we first removed SRPK1 from GST-pASF/SF2 using the following strategy. The phosphorylation reaction was mixed with 15 μl glutathione sepharose resin for 30 min at cold room. Beads were then washed sequentially with binding buffers (20 mM Tris-HCl [pH 7.5], 75 mM NaCl, 1 mM DTT, 2 mM MgCl_2_, 0.1% NP40, 10% glycerol, 1 mM PMSF, and 0.01 mg BSA) containing 2 M, 1 M, 0.5 M, 0.05 M, and 0 M urea. GST-ASF/SF2 and free GST were subjected to a similar treatment. Bound proteins were then put into reaction with 2 μg His-CLK1 in a cold room for 1 hr. Unbound proteins were removed by centrifugation and five washing steps using binding buffer with NP40 decreased to 0.5%. Bound proteins were separated by SDS-PAGE followed by western blot with anti-His and anti-GST antibodies.

### Crystallization

CLK1 was concentrated to 7 mg/ml in the presence of 10Z-hymenialdisine (0.5 mM final concentration) and buffered in 50 mM HEPES (pH 7.5), 500 mM NaCl, 5% glycerol, 50 mM L-glutamic acid, 50 mM L-arginine, and 10 mM DTT. Crystals were grown at 4°C in 150 nl sitting drops mixing 75 nl CLK1 with 75 nl of a precipitate solution containing 20% PEG 6K and 0.1 M bicine (pH 9.0). CLK3 crystals were grown at 4°C in 150 nl sitting drops mixing 75 nl CLK3 (11 mg/ml in 50 mM HEPES [pH 7.5], 200 mM NaCl, 10 mM DTT) with 75 nl of a solution containing 27% PEG 3350, 30 mM ammonium acetate, and 0.1 M BisTris (pH 5.5).

### Structure Determination

Diffraction data were collected on cryocooled crystals (100 K) using the X10SA beamline at the Swiss Light Source. CLK3 images were indexed and integrated using MOSFLM (Leslie, 1992), and scaled using SCALA (Evans, 1993) implemented in the CCP4 (CCP4, 1994) suite of programs. The CLK1 data were processed with HKL2000 (Otwinowski and Minor, 1997). The CLK1 structure was solved using molecular replacement and the program Phaser (Storoni et al., 2004) using human CDK6 (PDB code 1JOW) as a search model. CLK1 was used for molecular replacement in the determination of the CLK3 structure. Restrained refinement with TLS against maximum likelihood targets was carried out using REFMAC5 (Murshudov et al., 1997).

## Figures and Tables

**Table 1:** Structural Refinement and Data Collection

	CLK1	CLK3
Data Collection		

PDB code	1Z57	2EU9
Ligand	Debromohymenialdisine	None
Space group	C2	C2

Cell dimensions [Å]	a = 92.27, b = 64.43, c = 80.12	a = 108.72, b = 45.06, c = 84.01
α, β, γ	β = 119.19	β = 115.23

Resolution [Å]	1.7	1.53
Unique observations	45196	55697
Completeness^a^ [%]	95 (82)	99.8 (94)
Redundancy^a^	5.26 (1.77)	
*R*_merge_^a^	0.095 (0.25)	0.057 (0.37)

Refinement		

Resolution [Å]	60-1.7	60-1.53
*R*_work_ / *R*_free_ [%]	14.0 / 18.6	20.1 / 17.1
Atoms (P/L/W/)^b^	2692/ 18 /401	2848/ 0/ 347
B-factors (P/L/W/)^b^ [Å^2^]	21.3/ 13.9 /34	19.6/ — /29.1
Rmsd bonds [Å]	0.015	0.019
Rmsd angles [°]	1.591	1.743
Ramachandran		
Favored [%]	98.3	99.7
Allowed [%]	1.3	0.0
Disallowed	0.3	0.3

a Values in parentheses correspond to the highest resolution shell.

b (P/L/W) represents protein/ligand/water.

## Accession Numbers

Coordinates for the CLK1 and CLK3 structures have been deposited in the Protein Data Bank under the ID codes 1Z57 and 2EU9, respectively.

## Supplemental Data

Document S1. One Figure
