# Supplementary material for: Kinase Domain Insertions Define Distinct Roles of CLK Kinases in SR Protein Phosphorylation
Source: Structure. 2009 Mar 11;17(3-2):352–62. doi: 10.1016/j.str.2008.12.023 (PMC2667211; doi:10.1016/j.str.2008.12.023)

Structure *17*

Supplemental Data

Kinase Domain Insertions Define Distinct Roles of CLK Kinases in SR Protein Phosphorylation

Alex N. Bullock, Sanjan Das, Judit É. Debreczeni, Peter Rellos, Oleg Fedorov, Frank H. Niesen, Kunde Guo, Evangelos Papagrigoriou, Ann L. Amos, Suhyung Cho, Benjamin E. Turk, Gourisankar Ghosh, and Stefan Knapp

Figure S1.

Alignment and secondary structure of human CLK isoforms and SRPK1

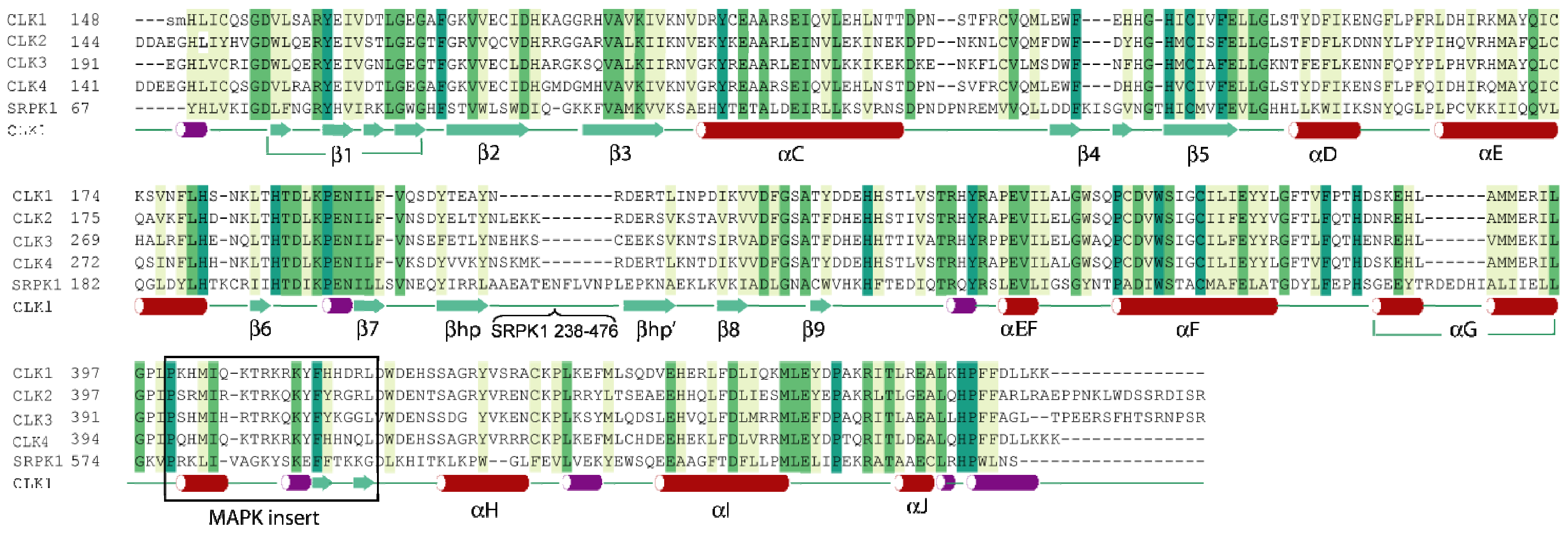

Supplement: Document S1. One Figure [file mmc1.pdf]
